# Key informant perspectives on sexual health services for travelling young adults: a qualitative study

**DOI:** 10.1186/s12913-022-07542-0

**Published:** 2022-02-04

**Authors:** Emmanuelle Gareau, Karen P. Phillips

**Affiliations:** Interdisciplinary School of Health Sciences, Faculty of Health Sciences, University of Ottawa, 25 University Private, Ottawa, Ontario K1N 6N5 Canada

**Keywords:** Travel, Tourism, Sexual health, Young adult, Health promotion, Sexually transmitted disease

## Abstract

**Background:**

International travel has become increasingly popular among young adults. Young adults often engage in casual sexual relationships abroad, exhibit sexual risk behaviours and may thus be at risk of contracting sexually transmitted and blood-borne infections. Pre-travel interventions and consultations may mitigate this risk. At present, we know little about sexual health-related pre-travel interventions. The aim of this study was therefore to document key informants’ experiences, perceptions and recommendations in the context of sexual health of young adult travellers.

**Methods:**

Key informants were professionals working in Ottawa, Canada travel clinics, travel organizations or sexual health clinics with a young adult clientele. This study used a qualitative approach and consisted of 13 in-person or Skype semi-structured interviews with key informants. Thematic content analysis was informed by a sexual health framework, with themes emerging both inductively and deductively.

**Results:**

Sexual health was not common in pre-travel interventions described by key informants. Risk-assessment, and practical or purpose-driven pre-travel interventions were identified, resulting in risk mitigation strategies tailored to the destination region and/or mission/culture of the travel organization. Dissemination (e.g. limited time, lack of training) and uptake (e.g. young adults’ embarrassment, provider discomfort, financial constraints) barriers limited in-depth discussions of pre-travel interventions related to sexual health. Key informants acknowledged the importance of early sexual health education, and recommended ongoing, comprehensive sexual education for both youth and young adults.

**Conclusion:**

The findings of this study suggest that more time and resources should be allocated to the topic of sexual health during pre-travel interventions with young adults. Professionals who guide and prepare young adults for travel must develop concomitant skills in sexual health promotion. Early, comprehensive sexual education is recommended to improve overall sexual health in young adults and mitigate risk behaviours during travel.

## Background

Youth and young adults, individuals between the ages of 15 and 24 [1], represent the greatest risk group by age for adverse sexual health outcomes such as sexually transmitted and blood-borne infections (STBBI), unintended pregnancy, sexual harassment/assault including misogyny and homophobia [2, 3] Transmission of STBBI and unintended pregnancies [2–4] in youth and young adults may be caused by inconsistent condom use [5, 6] high-risk behaviours such as drug and alcohol use, social media-related sexual encounters and multiple partners [7–10]. STBBI can contribute to many serious health complications such as reproductive cancers and infertility, and the presence of one or more symptomatic STBBI significantly increases the risk of HIV transmission [11–13].

Young adults are one of the most mobile generations in terms of international experiences [14]. In 2015, 23% of all international travellers were young adults (aged 15–29 according to the World Tourism Organization) [15]. Young adult travellers are more likely to spend long periods abroad, with purposeful travel - work, study, volunteer or language opportunities - steadily replacing leisure travel [15]. Motivations for international travel include interactions with local citizens and a desire for an immersive local, cultural experience [15]. Due to the COVID-19 pandemic, international travel is reduced significantly, including cross-border travel between Canada and the United States [16]. Vaccinations are gradually increasing Canadian international travel in 2021 [17], with normal travel volume anticipated post-pandemic.

The popularity of international travel has created a healthcare sector focused on the provision of travel-related health information [18, 19]. The field of travel medicine is predominantly preventative, ensuring pre-departure review, counselling and management of destination-specific risks, itinerary-specific risks, and risks specific to the individual traveller- such as pre-existing conditions [20]. Pre-travel health consultations typically include individual risk assessment, medical history, health risk education and related mitigation strategies specific to the travel region, as well as relevant immunizations and medications for prophylaxis and self-treatment [21, 22]. STBBI are among the communicable disease risks faced by travellers based on their destination or their planned activities [20]. It is difficult to quantify the impacts of travel on adverse sexual health outcomes due to the heterogeneity of travellers, trip characteristics and destination regions [23–27]. A systematic review estimated the prevalence of travel-associated casual sex as 20.4% with approximately 49.4% unprotected sexual encounters [27]. Risk factors for travel-acquired STBBI include casual sex, single status, alcohol or drug use, lack of travel advice, male, higher number of sexual partners and inconsistent condom use [23, 27]. Although discussion of sexual risk behaviours in pre-travel consultations by health practitioners is a best practice [20, 21, 28, 29] the quality and frequency of sexual health promotion is less well known.

Ottawa, Ontario is the bilingual (English, French) capital city of Canada, home to 934,243 residents including 125,840 young adults, aged 15-24 years [30]. Pre-pandemic Canadians were avid travellers, with 28.7 million international trips in 2010; 44.6% representing travel by Ontario residents [31]. Although the United States remains the most popular destination, Canadian travellers also include overseas backpackers, volunteers traveling to low-income countries, and immigrants returning in their country of origin to visit relatives [32]. Canadian Generation Z (age 18-23 y) travellers prioritize experiential travel that includes physical activity, exploration and inspiration [33]. Given the increased risk of adverse sexual health outcomes, including STBBI, unintended pregnancy, and sexual harassment/assault, among travelling young adults, travel health interventions can be considered an essential component of public health. This study aims to investigate the experiences, practices and recommendations of Ottawa-region professionals, employed in fields of travel medicine, sexual health or travel/tour agencies, within the context of young adult travellers’ sexual health.

## Methods

### Sample

Recognizing that young adult travel may include pre-departure consultations with both travel organizers, and travel health providers, EG recruited travel health sector key informants to participate in semi-structured interviews through purposive sampling by email, phone or in person. To best capture an overview of the travel health sector, interviews with key informants convey, by proxy, the experiences, practices and culture within their respective organizations [34, 35]. Inclusion criteria for key informants were as follows: professionals employed in the travel sector within the Ottawa region who routinely provided pre-travel health counselling and guidance to young adults, and were able to participate in the interview in English or French. Lack of English/French language proficiency, organizations which did not offer services in the Ottawa-region, and professions limited to travel/itinerary arrangements (e.g. travel agents) were exclusion criteria. Thirteen key informants from 11 different organizations, including travel-health clinics, sexual health clinics, young-adult-focused tour agencies and health-promotion groups, with no restriction on the type of organization/business (e.g. commercial, health, academic, non-governmental organization) were recruited. Given that key informants were drawn solely from organizations based in the Ottawa-region, and that there are limited such travel services which provide guidance and counselling related to health and wellness, this sample size was deemed adequate [36] to reflect a diverse breadth of local organizations and upon data analysis, saturation was achieved [37].

### Data collection

The interview guide was informed by the World Health Organization’s sexual health conceptual framework [38] which promotes sexual health as the holistic achievement of physical, emotional, mental and social well-being, framed by human rights, evidence-based and a sex-positive approach. Interview topics included: Key informants’ (i) roles in travel interventions related to sexual health, (ii) perceptions of young adult Ottawa-region travellers’ sexual health and experiences, (iii) practices and recommendations for travel-related sexual health promotion; (iv) challenges and barriers to sexual health promotion; and (v) demographics.

### Data analysis

Interviews were audio-recorded, transcribed and analyzed in the original source language, with field notes confirming that thematic saturation was reached [39, 40]. Translations of sample quotations from French-speaking participants appear in this article. Interview transcript data, coded using NVivo (Version 11.4.3 (2084)), were analyzed using both inductive [39, 40] and deductive approaches; supported by the WHO sexual health framework [38] and the interview guide. EG analyzed the data iteratively and the research team reviewed and discussed the interpretation and qualitative content analysis [39] to fully develop our understanding of the data.

### Ethics

All methods were performed in accordance with relevant medical ethics and research guidelines and regulations. All study protocols were reviewed and approved by Research Ethics Board at the University of Ottawa (File # H02-17-14). EG conducted English and French interviews in person or via Skype, between April-November 2017, with seven English- and six French-speaking participants, each of whom provided written, informed consent to participate in the study.

## Results

### Participant characteristics

Key informants included seven professionals working in a health-related organization (4 travel clinics, 3 sexual health clinics) and six employed by travel services (Table 1). The majority of the key informants identified as female (*n* = 11) and had less than 10 years of experience in their current field of work (*n* = 8). Recognizing that mandates of healthcare (i.e. travel clinics, sexual health clinics) are distinct from travel services (i.e. organizations specializing in international cooperation, tourism, volunteering or student exchanges), our presentation of the findings notes major differences between these groups.

**Table 1:** Participant Demographics

Characteristic	Sample
**Gender**	
Female	11
Male	2
**Age**	
25-34	5
35-50	4
+ 50	4
**Highest Educational Attainment**	
Bachelor Degree	7
Post-Graduate Degree	6
**Professional Role**^**a**^**/Title**	
Public Health Nurse (RN)	5
Physician (MD)	1
Non-Clinical Administrators^b^	7
**Years of Experience in Current Field**	
< 5	6
6-10	2
11+	5

^a^*RN* registered nurse, *MD* medical doctor

^b^Non-Clinical Administrators- includes program directors, managers, recruitment agents, team leaders

### Pre-Travel interventions

In general, key informants described pre-travel interventions tailored to the mission of the tour agency or the destination region, with sexual health not considered a core component. Themes related to informants’ pre-travel practices emerged as either *risk-assessment approach* and/or *practical or purpose-driven interventions*. Healthcare key informants typically used risk-assessment approaches to mitigate disease risk or potential health hazards (e.g. food, water, mosquitoes, etc.) associated with the destination region. Typical pre-travel healthcare interventions included vaccinations, medications and general travel advice. When asked how young travellers’ sexual health could be improved, a female travel nurse (EG01) said, *“[…] we have to focus on vaccines and medications like malaria- we have to focus on the risks of illness,”* following the guidelines and directives of the host organization. A travel services informant recognized the risk-assessment approach used by healthcare workers, and recalled that the nurses responsible for the pre-travel health module training emphasized risks related to diseases:

*“It’s really only an overview. Because whether you want it or not, in the pre-departure training, we have to talk about so many other diseases, parasites, this and that. So it* [STBBIs] *are rarely discussed. You know, we won’t go into details.”* [translation]; -male, program officer, travel services, EG11

Travel services participants reported that most humanitarian or volunteer opportunities are organized as group activities. Although practices varied across different host organizations, informants used practical or purpose-driven interventions tailored to the collective needs of the group. Travel services key informants noted little emphasis on sexual health in the pre-travel health discussions.

“*We have only one slide on* [sexual] *health. What is said is things like ‘don’t forget your condoms’ or ‘if you are a woman and are on the pill, talk with your family physician and ask them for a prescription and make sure you have enough’. We tell them ‘condoms are not always available in all sizes in all different countries, so bring what you need’. They’ll find that funny, but at least, we tell them.*” [translation]- female, program director, travel services, EG02.

Participants from travel services perceived that healthcare professionals were better able to address sexual health topics, with pre-travel health interventions outsourced to travel nurses/medical clinics.

“*It’s not our responsibility to do their sexual education necessarily, but we remind them that* [sex] *isn’t the same abroad, not everything we have here is available abroad, that if they need prescriptions, they need to think about that, to put it on their to-do list. I think it’s part of our responsibility, but I’m not sure that I have the right competence to go in depth* [...] *We don’t focus on health”.* [translation], female, program director, travel services, EG02.

Although sexual health was most often discussed in terms of casual sex, participants were cognizant that adverse sexual health outcomes could include harassment and assault. The risk-assessment approach was used to discuss travel-safety, which both health and travel services key informants discussed in terms of acceptability of sexual expression and risks of sexual assault.

*“…we know how difficult it is for people to talk about.. um.. sex in general, sexual assault and harassment in particular, is even more taboo and difficult, so if we talk to them before them* [sic] *leave, and we tell them that there is a policy and there are procedures and we take this extremely seriously, then when it does occur um- and the percentage of it occurring are quite high …, they’ll be more likely, one, to report it if um they’re the victims of harassment or assault, and two, to be more aware of it happening in their environment”.* female, program director, travel services, EG07

*“Our patients- it’s really to talk about safe sex, uh, we will discuss if they’re travelling to a country where there’s a high incidence of HIV. … I talk about, for young girls, especially travelling to certain places where it might be dangerous for them, that there has been cases of rape, even, kidnapping in things like group rape”-* female, nurse, EG03

*“In fact, we talk about sexual harassment, a lot from a work perspective, we also talk about homosexual relations, which for us too is a major issue because in many countries where we operate ... homosexuality, it is either prohibited or criminal, or otherwise it is taboo. Some countries even, it's illegal.”* [translation] male, program officer, travel services EG11

Key informants from volunteer-travel services mentioned that no-sex contracts or no-sex policies for travellers were quite common, although such abstinence-policies were rarely enforced in the field.

*“Somebody told me that like years ago, there used to be a message, like* ‘no sex is best’*, and they said that was so silly because people are gonna do what they wanna do, so you should be telling them more, you know, how to protect themselves in the decisions they make.”-* female, program director, travel services, EG07

*“In our first aid kit, we have condoms and Plan B* [morning after pill]*. Still, it* [having sexual relationships]*'s forbidden. They have signed a contract that says they can’t have sex, whether it’s sex with locals or with people from the groups. You know, we are pretty clear on that. But we could give condoms and Plan Bs. You know, at some point, they are adults and we know it can happen. So we tell ourselves that if it happens, we can deal with it*” [translation] – male, program officer, travel services EG11

### Barriers to sexual health promotion

Major themes related to key informants’ pre-travel sexual health promotion were *dissemination* and *uptake barriers*. Dissemination barrier subthemes included; (1) limited time for discussion, (2) lack of suitable resources (i.e. sexual health training), and (3) perceptions that sexual behaviours of adult travellers are private.

“*In terms of travel, because you’re seeing sometimes someone for the first time, and there’s a lot of things you need to cover. Because they’re more concerned about what vaccines they need, right? There’s a lot to cover so…I mean, I would say the sexual health part kind of gets squeezed a bit…just for time. But, I mean, I always make the attempt to talk about it*”- male, travel physician, EG08

“*[…] it’s time limited, and there are many clinics, or speakers, who I know who will say that, you know, we need to incorporate this* [sexual health] *into our consultations. But it’s easier said than done. I think having written information for clients to take home, that’s really concise, and full of information would be much more beneficial*.”- female, travel nurse, EG01

*“Sexual health is important for sure. But we consider that they* [travellers] *are adults, they are university students and they know a little bit about that stuff.”* [translation] *-*female, program director, travel services, EG02.

Uptake barriers related to the accessibility of sexual health promotion itself by young travellers. Barriers to sexual health uptake by travellers were categorized as three subthemes: *(1) young adults’ embarrassment, (2) travel/healthcare professionals’ discomfort*, and (3) *travellers’ financial constraints*. Both travel services and healthcare respondents described young travellers’ discomfort with sexual health discussions, perceived shyness, apparent disinterest, or requests to change topic when sexual health was introduced.

“*I think they’re also very shy. People are just generally shy. And not comfortable speaking about sexual health.”* -female, travel nurse, EG01

“*Some it seems like they’re just coming in for that vaccine... it’s to get their attention to talk about everything else, but you’ll see their eyes glaze over and you know they’re already thinking, or they’re on their phone texting somebody, and they think that the visit’s over*.” -female, travel nurse, EG03

“*We have to just judge from their response, whether they’re open to it or not, right? You know, some say, ‘I know, I know, you don’t have to tell me’, like that- then obviously, I’m not gonna go into it too much.”* -male, travel physician, EG08

Key informants considered that although some young adults overestimate their personal knowledge about sexual health, others are uncomfortable talking about sex, especially if there are no plans to have sexual relationships while abroad. A travel services participant perceived that young adults feel condescended to when the topic of sexual health is discussed.

*“Often they are paternalized. Often, it’s a bit like you impose a decision on them…Sometimes, youths think they know a lot. We have the feeling that they have an ‘*I do not care*’ attitude. They* [youth] *are like ‘*Who are you to tell me that kind of things?*’”* [translation]-male, program officer, EG11.

Respondents also acknowledged that travel/healthcare professionals often feel uncomfortable discussing sexual health, thereby contributing to the uptake barrier.

*“I don’t think there are a lot of family doctors or travel clinics who are comfortable talking about this* [sexual health; translation].” *-* female, sexual health nurse, EG04

A travel nurse mentioned that her employer refused to talk about sexual health topics and referred his patients to her for sexual health-related questions:

“*That’s why the physician didn’t speak to you* [EG]*; he doesn’t feel as comfortable. Now he’s an older gentleman, it’s a different generation. He doesn’t like to bring up the sexual aspect of the visit. So I think it’s a matter of even healthcare professionals feeling comfortable enough to discuss it without feeling embarrassed or shy, or feeling like you’re imposing your opinion. But you have to put yourself outside of it somehow and be comfortable to just give factual information, in a way that they’re gonna take it in*.” -female, travel nurse, EG03.

A humanitarian travel organization key informant seemed ill at ease when discussing sexual health topics during the interview,

*“I don’t think it’s* [sexual health] *a common topic. I don’t know if it’s because the organization might think that talking about it might encourage people…encourage* [sexual] *actions…For us, it’s difficult…We are all together during fifteen days in the same environment…I don’t think it’s appropriate.”* [translation] -female, travel services mission lead, EG13

Respondents also considered young travellers’ financial limitations as barriers to their uptake of sexual health information, given the consultation fees required for some travel-health services.

*“Many times, young adults- because of cost issues and finances- may- sometimes will come in, and only- they know they only want Hepatitis A and B. That’s it. Sometimes they don’t want to hear about anything else. Sometimes they only want uh what’s required. And the only thing that is required can be Yellow fever vaccine. There’s nothing else that’s required to go uh into certain countries. So, there are times where they don’t want to listen to a consult, they don’t want all the information; but mostly, it’s because of budget. They have a small budget, and they want the bare necessities… our hands are tied there. Our cost is what it is. Um, we cannot give free vaccines; we cannot lower the prices of our vaccines.” .* […]*” -*female, travel nurse, EG01

*“The cost, as in the services we give have a cost behind it. It’s not covered by OHIP* [Ontario Health Insurance Plan] *if it’s travel. If they’re coming in for their booster, their visit it’s covered by OHIP, but otherwise they’re paying for their services here”*- female, travel nurse, EG03

*“You never have enough time to get young people- certain young people comfortable enough to start talking and to get really engaged with them. And when you just start to get really close to them, really talking outward and honest with you, and displaying their needs and their issues, and getting questions, their time is up, and you don’t get enough time with them. And our services cost, so it’s not like they can just keep coming back and back and back, and taking all that time. They can’t afford that!”* -female, travel nurse, EG09.

### Sexual behaviours and travel

An overarching theme which emerged across interview questions, was the recognition by both health and travel key informants that travel itself may cause young adults to engage in risky sexual behaviours. Key informants’ perceptions of the impact of travel on sexual behaviours were summarized as the following subthemes: *disinhibiting effects of alcohol, anonymity of travel*, and *exoticism of travel*.

Travel-related increased alcohol intake was widely acknowledged as interlinked with casual sex.

*“Well, I think we all know that alcohol and sex go to together ... When you’re away, um you will drink- most people will drink more…. where there’s a lot of drinking involved, they’ll absolutely be at a higher risk of sexually transmitted diseases and probably more frequent unplanned sex.”-* female, travel nurse, EG01

*“You know, students, when they travel abroad, they party. Often, they party a lot and they meet people and they are more vulnerable”* [translation]- female, program director-travel services, EG02.

The anonymity of travel, being away from social networks at home, was recognized as a contributing factor to increased sexual activity abroad. A volunteering program officer explains:

“*You’ll keep your anonymity and you will do more things…and this is the danger that we often witness. You have less pressure, or at least, the pressure you feel is different. While travelling, you’ll do things that you wouldn’t necessarily do. There’s the exotic side of things. People always have risky behaviours, but while abroad, I’ll say that 10-15%, even 10-20% of travellers have problematic risky behaviours. You allow yourself to do more things because you’re just passing through the country. You’re more nonchalant.”* [translation] -male, program officer, travel services, EG11

“*Travellers take more risks. There is a lot of infidelity. People travel to have sexual encounters and be unfaithful, because it’s less likely that you’ll have to deal with the repercussions. If they are at home in Ottawa and want to have a one-night stand, casual sex, there are more risks involved that people may see you or that you see your one night partner again. I think that people find it easier to go on a trip and be anonymous. We see a lot of that*” [translation]- female, sexual health nurse, EG04

Anonymity is undoubtedly part of the exoticism of travel, but also perceptions that restrictions, obligations and rules are left behind while on vacation. Key informants acknowledged that pre-travel sexual health promotion was necessary to challenge the “what happens in Vegas stays in Vegas” culture frequently embraced by young adults.

*“[…] people will behave differently on vacation. And maybe throw caution to the wind more”* -female, travel nurse, EG03

“*I’d say that the sexual health of young adults travelling is more at risk as people are in a new context, one of adventures, new experiences and discoveries and because of this thrill they feel, will be less cautious, especially when they just arrived.*” [translation] -female, volunteering organization recruitment agent, EG12

“… *you know the famous notion of when you are abroad, anything goes. Then I think you start to associate it with a lot more abroad than at home…”* [translation] -male, program officer, travel services, EG11

A healthcare professional considered that many young adults may return from travel and reflect on their travel-related sexual behaviours with regret, health or safety concerns resulting in “post-travel anxiety”.

*“People will travel, come back and only then will the reality strikes. They’ll be very, very anxious and they’ll have regrets about behaviours they had or things that happened. There will be a lot of anxiety like “what have I done”, anxiety towards STIs, towards* [STI] *testing, things like that. We often see anxious people, coming back from a trip, who have regrets and* fears [concerning their sexual health; translation],*” -*female, sexual health nurse, EG04

### Key informant recommendations

When asked to provide recommendations related to sexual health and travel, several themes emerged about key informants’ general perceptions of young adults: early sexual health promotion and education is a determinant of adult sexual health and well-being; young adults exhibit a general lack of sexual health knowledge, and comprehensive sexual education targeted to youth and young adults is the recommended strategy to improve these knowledge gaps.

“*We can obviously do more because we have such a high rate of sexually transmitted diseases, so obviously we’re not doing our job. For sure, there could be more done and more taught. Starting from maybe making it easier taught in schools, but they do talk about it. ….. I do believe that there is a lot of ignorance and a lot of young people, they still think that they are invincible. It only happens once, or it’s not going to happen to me.” -*female, travel nurse, EG01

*“I think there’s this idea still that maybe a lot of us* [young adults] *are invincible, and that it happens to other people. And I think there’s a lot of myths and misinformation when it comes to health. Definitely.” -* female, sexual health counsellor/coordinator, EG06

## Discussion

We report here that sexual health is generally omitted, discussed superficially, or considered an awkward, uncomfortable topic by Ottawa-region key informants. Respondents recognized that the anonymity and exoticism of travel, along with use of disinhibiting substances like alcohol and drugs increased travel-associated sexual behaviours among young adults. Healthcare key informants prioritized risk-assessment driven interventions, emphasized infectious diseases other than STBBI, prophylactic vaccinations and general health and safety; whereas travel services participants relied on practical or purpose-driven interventions tailored to the immediate needs and activities of the travellers. Key informants identified barriers to the dissemination of sexual health promotion but also perceived that uptake of such messaging was impeded by provider/young adult discomfort and embarrassment with the subject.

A 2016 systematic review identified that less than half of travellers from high income countries received pre-travel sexual health interventions [23]. Previous studies report that sexual health risks are rarely discussed in pre-travel consultation in travel clinics [41, 42], consistent with our findings. Although pre-travel sexual health promotion is a recommended best practice for travel medicine [20], evaluation of the benefits of these interventions is complicated by inconsistent, non-standard programmes, heterogeneity of both providers and travellers and trip destinations [27, 32]. Whereas some studies conclude that pre-travel sexual health promotion can be effective in reducing adverse outcomes [23, 42], other studies consider that such pre-travel sexual health interventions may successfully increase sexual health knowledge and risk perception without necessarily reducing risk behaviours [43, 44],

### Reported barriers to sexual health promotion

Reported uptake barriers of sexual health promotion during pre-travel consultations include format and perceived relevance of the sexual health travel-related promotion [24, 25]. In our study, travel and health professionals articulated discomfort, lack of training and confidence associated with sexuality discussions as barriers. This is consistent with implementation barriers for sexual education by teachers [45, 46], and clinical sexual histories/counselling by medical students [47, 48], and nurses [49, 50]. Training and continuing education are strategies to enable sexual educators, including health professionals, become more assertive and confident with sexuality discussions [46, 49]. Attitudes related to perceived discomfort and embarrassment of clients, students, patients, and in our study, young travellers, often reflect gaps in understanding of the importance of sexual promotion to the target audience, insufficient training or other barriers including lack of time or privacy [46, 49]. Training by itself, however, cannot abrogate policies, protocols or organization cultures which do not support open, inclusive sexual health promotion [45]. Best practices for sexual health promotion should ensure a judgment-free dialogue that is sex-positive [38, 46, 51], tailored and relevant to the audience, and is evidence-based [1, 38, 52]. Although sexual health promotion may provide comprehensive discussion about risk reduction, and safe sex, broader issues related to sexualities, sexual rights and gender equity are often omitted [53]. Inflexible personal beliefs about sexualities may bias sexual health promotion, contribute to missed opportunities for needed referrals, information and counsel, and lead to incorrect sexual health risk assessments [47]. Therefore, an essential starting point for inclusive, sex-positive sexual health promotion is relevant training and personal reflection for providers, so that they are able to meaningfully discuss these issues with trust, respect and dignity.

A cis-heteronormative lens of sexuality does not meaningfully reflect diverse sexual identities in Canada [46], such that the needs of minority groups may go unaddressed with respect to sexual health and travel. Travel key informants discussed sexual harassment, risk of sexual assault and cultural/legal acceptability of homosexuality and non-binary sexual identity expression abroad, however these topics received less emphasis compared with standard pre-travel consultation topics (e.g. food and water safety, medication, culture shock). Key informants framed gender-based violence along with legality of sexual expression abroad as human-resource or safety topics, rather than part of the broader dialogue about sexual health. Responsibility for sexual violence always rests with the perpetrator, however risk behaviours such as travel destination, excessive intoxication, accommodation, nightlife activities, and solo travel, are associated with assaults on young women and gay and bisexual men [54, 55], Tourism-sector workers face considerable sexual harassment, such that young adult travellers may also represent potential perpetrators [56]. It was unclear the intent of established abstinence policies and no-sex contracts for travellers described by Ottawa-travel services informants. These policies may serve to reduce risk of sexual assault, and possible legal repercussions of casual sex abroad, and may also be intended to safeguard the reputation of participating travel organizations by minimizing problematic sexual behaviours of travellers abroad. Key informants acknowledged that these abstinence contracts were difficult to monitor and enforce.

### STBBI

Healthcare key informants identified that the topic of vaccines, especially hepatitis B vaccine, was used as a mechanism to briefly introduce sexual health, mainly HIV and STBBI, in pre-travel interventions. Emphasis on travel STBBI risk reduction is essential, as globalization and travel have facilitated STBBI dispersion and impacted surveillance and treatment [23, 27, 32, 44]. Indeed, travellers who engage in sexual relationships abroad are exposed to new and different sexual networks [21] especially when travelling to STBBI hot-spots [57]. Travel-related sexual networks can facilitate the introduction of new drug-resistant STBBI in the home country, including drug-resistant strains of *N. gonorrhoea* [21, 27, 44], as well as other blood-borne pathogens [27, 32] like the Zika virus [58, 59]. As young travellers, particularly men, are at higher risk to engage in casual sexual relationship abroad [21, 26, 27], it is important that pre-travel consultations address the issue of STBBI and safe sex abroad [21, 60].

### Recommendations

Our findings indicate that Ottawa-region travel and health key informants provide only limited sexual health pre-travel interventions. Most international health organizations identify sexual health is as an essential pre-travel intervention (e.g. CDC [21], International Society of Travel Medicine (ISTM) [29], Committee to Advise on Tropical Medicine and Travel [CATMAT] [28]). Further, travellers should be encouraged to follow up with post-travel interventions, including sexual health assessments. It is evident that Ottawa-region travel services prioritize pre-travel preparations, rather than post-travel follow-up or referrals. Health key informants were more likely to manage post-travel incidents, however there did not appear to be a systematic scheduling of post-travel follow-up appointments. Post-travel interventions to improve follow-up could include automated reminders issued from travel organizers, airlines or public health agencies.

More research is needed to identify the best practices in terms of pre-travel health interventions and sexual health information uptake by young travellers [61]. Young adults perceive health hazards, such as unprotected sex, as less risky, compared with older adults [62]. Dialogues about sensitive topics such as sexual health with adolescents and young adults may create discomfort for both provider and patient [63], consistent with the views expressed by our key informants. For providers, sexual health training, youth/young adult-targeted sexual health guidelines, and sexual risk assessment decision aids, such as the touch-screen-administered measure Sexual Risk Behavior Inventory (SRBI) for primary care patients [64], may help instill confidence and ability. Engagement of adolescents/young adults in such discussions requires establishment of trust and emotional comfort initiated by informal conversation [63]. Our health key informants seemed to realize this, however, they described very little time to establish patient rapport, with consultation time restricted to priority travel vaccinations. Once trust and rapport are established, provider-guided interventions that are sex-positive, individually-tailored could include condom negotiation, safer, alternative non-penetrative sexual practices such as mutual masturbation [43, 65]. Digital mechanisms to deliver sexual health information and recommendations should be used to complement classic provider-interventions. Use of digital apps such as *GGD Op reis* [66], *MyTravelHealth* [67] or CDC’s app *TravWell* [68] and travel SMS/text messages with sexual health promotion [69] may better serve this young adult demographic. Although we recommend modernization and diversification of pre-travel sexual health promotion platforms, these mechanisms should complement face-to-face provider consultations which can best provide individualized counsel and provision of evidence-based advice, prophylaxis/mitigation immunizations and medications [70].

## Limitations

This qualitative study is not meant to be generalizable or representative of the experiences and practices of all key informants working in travel clinics, sexual health clinics and travel organizations. Several organizations declined our invitation to participate in the study; citing lack of coverage of sexual health topics or discomfort with the topic. This suggests a sampling bias, as the key informants who agreed to participate did address sexual health to some extent or were comfortable discussing this topic.

## Conclusion

The findings from our study highlight that Ottawa-region health or travel key informants omit or neglect sexual health in pre-travel interventions. We recommend that youth-focused travel organizations emphasize within pre-travel consultations for comprehensive sexual health discussions that recognize the intersectionality of sexual identities, race/ethnicity, gender, age and citizenship. Both travel and health key informants should consider established and emerging sexual risk assessment decision aids to facilitate sensitive discussions and use of digital technologies for efficient, cost-effective sexual health promotion at the population level.

## Data Availability

The data (interview transcripts) that support the findings of this study are in the posession of authors (Corresponding/KP Phillips), but restrictions apply to the sharing of these data, and so are not publicly available. Only publishing/dissemination of quotations was approved by University of Ottawa Research Ethics Board, not the entirety of original interview transcripts. Similarly, study consent forms to participate in interviews only authorized researchers to use selected quotations not entire transcripts for publication/dissemination. Selected quotations appear throughout the article, reproduced with permission.

## Abbreviations

CDC: Centers for Disease Control and Prevention
HIV: Human immunodeficiency virus
SRBI: Sexual Risk Behavior Inventory
STBBI: Sexually transmitted and blood borne infections
STI: Sexually transmitted infections
WHO: World Health Organization
